# The impact of COVID-19 and socioeconomic status on psychological distress in cancer patients

**DOI:** 10.1016/j.ijchp.2023.100404

**Published:** 2023-08-22

**Authors:** Elisabeth Lucia Zeilinger, Matthias Knefel, Carmen Schneckenreiter, Jakob Pietschnig, Simone Lubowitzki, Matthias Unseld, Thorsten Füreder, Rupert Bartsch, Eva Katharina Masel, Feroniki Adamidis, Lea Kum, Barbara Kiesewetter, Sabine Zöchbauer-Müller, Markus Raderer, Maria Theresa Krauth, Philipp B Staber, Peter Valent, Alexander Gaiger

**Affiliations:** a Division of Hematology and Hemostaseology, Department of Medicine I, Medical University of Vienna, Vienna, Austria; b Academy for Ageing Research, Haus der Barmherzigkeit, Vienna, Austria; c Department of Internal Medicine, Landesklinikum Baden-Mödling, Baden, Austria; d Department of Developmental and Educational Psychology, Faculty of Psychology, University of Vienna, Vienna, Austria; e Division of Palliative Medicine, Department of Medicine I, Medical University of Vienna, Vienna, Austria; f Division of Oncology, Department of Medicine I, Medical University of Vienna, Vienna, Austria; g Ludwig Boltzmann Institute for Hematology and Oncology, Medical University of Vienna, Vienna, Austria

**Keywords:** Cancer, COVID-19, Psycho-oncology, Socioeconomic status

## Abstract

**Objective:**

We aimed to investigate the impact of the COVID-19 pandemic on psychological symptom burden against the socioeconomic background of cancer patients using data from routine assessments before and during the pandemic

**Method:**

In this cross-sectional study, standardised assessment instruments were applied in *N* = 1,329 patients to screen for symptoms of anxiety, depression, post-traumatic stress, and fatigue from 2018 to 2022. Two MANOVAs with post-hoc tests were computed. First, only time was included as predictor to examine the isolated impact of the pandemic. Second, income level and education level were included as further predictors to additionally test the predictive power of socioeconomic factors

**Results:**

In the final model, only income had a significant impact on all aspects of psychological symptom burden, with patients with low income being highly burdened (partial η² = .01, p = .023). The highest mean difference was found for depressive symptoms (*MD* = 0.13, *CI* = [0.07; 0.19], *p* < .001). The pandemic had no further influence on psychological distress

**Conclusions:**

Although the pandemic is a major stressor in many respects, poverty may be the more important risk factor for psychological symptom burden in cancer outpatients, outweighing the impact of the pandemic.

## Introduction

The COVID-19 pandemic had and still has a significant impact on mental health in various groups of people all over the world (Lafta & Mawlood, 2022; Maunder et al., 2021; Roy et al., 2021). People with a cancer diagnosis are at particular risk of developing comorbid mental disorders (Zeilinger, Oppenauer et al., 2022) that not only affect quality of life, but also have a significant impact on physical outcomes and survival (Gaiger et al., 2022; Knefel et al., 2023; Unseld et al., 2021).

Psychological distress in cancer patients might have further increased during the pandemic (Islam et al., 2021). The need for mental health care and counselling was reported to be “skyrocketing” (van de Haar et al., 2020). However, studies on mental health problems in cancer patients during the pandemic report mixed results (Ayubi et al., 2021). While some studies found elevated mental health problems, including symptoms of depression and anxiety (Ernst et al., 2022; Islam et al., 2021), others found no increase in psychological symptom burden (Rentscher et al., 2021; van de Poll-Franse et al., 2021). Studies about the impact of the COVID-19 pandemic on mental health in the general population also come to varying conclusions (Brunoni et al., 2021; Hajek et al., 2022).

For people who were more directly affected by the pandemic, such as health care workers, research suggests that the pandemic did indeed increase psychological distress and emotional exhaustion (Maunder et al., 2021; Mehta et al., 2022; Shechter et al., 2020). In cancer patients this effect has not yet been conclusively established. One shortcoming of previous research on cancer patients is the exclusive use of data collected during the pandemic, which cannot be reliably compared with the pre-pandemic period. Cancer patients commonly show high rates of psychological distress (Zeilinger, Oppenauer et al., 2022). Without an appropriate reference period before the pandemic, these high rates cannot be attributed to the pandemic. Furthermore, sociodemographic factors may mediate the mental health response of cancer patients during the COVID-19 pandemic.

In Austria, the population was confronted with four lockdowns to contain the spread of infection, which had a massive impact on the labor market. The Viennese population showed the highest unemployment rate during the pandemic, increasing from 10.0% in 2018 to 12.1% in 2021, in line with the national trend during this period (Statistik Austria, 2022). The situation for cancer patients in Austria was initially problematic: from March to May 2020 (first wave with lockdown) the number of hospital stays due to a cancer diagnosis decreased by up to 20% (GÖG, 2022). Although the care situation returned to normal afterwards, cancer patients continued to report an increased fear of infection during hospital treatment (Gerger, 2021). The outpatient clinic, where our data were assessed, remained open throughout the pandemic to any patient who wished to come in for an examination or treatment.

Recent research found that socioeconomic status (SES) interacts with individual response to both containment measures’ extension and ending (Serrano-Alarcón et al., 2022), and to healthcare seeking behavior (Zeilinger, Lubowitzki et al., 2022). Our research objective was to investigate the impact of the COVID-19 pandemic and of socioeconomic status on psychological symptom burden of cancer outpatients by comparing data routinely collected before and during the pandemic.

## Material and methods

We have followed the STrengthening the Reporting of OBservational studies in Epidemiology (STROBE; Elm et al., 2007) guidelines in our reporting.

### Sample

The total sample comprised 1329 outpatients with cancer or other hematologic neoplasms (49.6% female). Age ranged from 18 to 92 years (*M* = 59, SD = 14.14). The most prevalent diagnosis in our sample was hematologic cancer/neoplasms, followed by lung cancer and breast cancer. The sample included 636 patients in the reference timeframe two years prior to the COVID-19 pandemic (from Mar 2018 to Feb 2020), and 693 within the first pandemic years (from Mar 2020 to Jun 2022). Socio-demographic and clinical characteristics of the sample are depicted in Table 1.

**Table 1:** Socio-demographic and clinical characteristics of the sample.

Characteristic	*n*	%
Gender		
Female	659	49.6
Male	670	50.4
Marital status		
Single/widowed/divorced	484	36.4
Married/partnered	845	63.6
Educational level		
Primary education/apprenticeship	489	36.8
Secondary education	448	33.7
Postsecondary/tertiary education	392	29.5
Monthly net household income		
<1300 Euro	270	20.3
1300 – 2200 Euro	418	31.5
>2200 Euro	641	48.2
Employment		
Employed	616	46.3
Unemployed	94	7.1
Retired	619	46.6
Cancer type		
Haematologic cancer/neoplasms	324	24.4
Lung	167	12.6
Breast	132	9.9
Soft tissue	92	6.9
Head and neck	73	5.5
Colon/Rectum	73	5.5
Pancreas	68	5.1
Brain	64	4.8
Stomach/Oesophagus	51	3.8
Kidney/Urinary tract/bladder	38	2.9
Melanoma	29	2.2
Female genital organs	21	1.6
Prostate	21	1.6
Hepatobiliary	19	1.4
Thyroid	12	0.9
Other	145	10.9

Note. *N* = 1329.

### Procedures

Data for this study were assessed at the hematological and oncological outpatient clinic of the Vienna General Hospital from 2018 to 2022. The following inclusion criteria were applied: (1) confirmed diagnosis of cancer or other hematologic neoplasms, (2) age ≥ 18, (3) capacity to consent, (4) sufficient German-language skills. After explanation of the study and written informed consent, patients were handed out questionnaires. The response rate was 78%. Reasons for non-participation given by patients included not having enough time to complete the questionnaire or not wanting to be bothered with a study. The study was approved by the institutional ethics committee of the research site (EC Nr: 2255/2016; 1241/2021).

### Materials

Data was collected by means of a questionnaire that is part of the routine assessment in our outpatient clinic. This questionnaire consisted of a sociodemographic profile, including the SES indicators monthly net household income and educational level, and standardized assessment instruments, namely the Hospital Anxiety and Depression Scale (HADS; Zigmond & Snaith, 1983), the Post-Traumatic Symptom Scale (PTSS-10; Stoll et al., 1999), and a visual analogue scale (VAS) to assess fatigue.

#### Hospital anxiety and depression scale

The HADS is a 14 item self-report screening tool with seven items each relating to anxiety and depression (Zigmond & Snaith, 1983). Psychometric evaluations indicated good results in cancer patients (Zeilinger, Nader et al., 2022). All items are rated on a 4-point Likert scale ranging from zero to three. Two scores can be calculated: the depressive symptoms score (HADS-D) and the anxiety symptoms score (HADS-A). Higher scores represent higher symptom burden. Scores up to seven indicate no depression/anxiety, scores between eight and ten imply a possible anxiety/depressive disorder, and scores higher than ten indicate significant depressive/anxiety symptoms. Internal consistencies (McDonald's omega (McDonald, 1999)) in the present study sample were high, with omega = 0.87 for the HADS-D, and omega = 0.85 for the HADS-A.

#### Post-Traumatic symptom scale

The PTSS-10 is a ten item self-report instrument to assess post-traumatic stress symptoms (PTSS). Each item is rated from zero to three, with higher scores indicating higher symptom burden. A total score higher than 12 implies significant PTSS. Psychometric evaluations indicate that the PTSS-10 is a responsive, valid and reliable screening tool for PTSS (Stoll et al., 1999). Internal consistency in the present study sample was high, with omega = 0.87.

#### Visual analogue scale

Fatigue was measured on a one-item VAS ranging from zero to ten. This assessment method was shown to be feasible and valid in cancer patients (Temel et al., 2006), with even higher sensitivity and reproducibility than a Likert scale (Grant et al., 1999).

### Statistical methods

Two multivariate analyses of covariance (MANOVAs) were computed. For both, dependent variables were the scores of depression, anxiety, post-traumatic symptoms (PTSS), and fatigue. In the first analysis, we investigate solely the impact of the COVID-19 pandemic on psychological burden of cancer patients, with time of assessment included as predictor. We used a reference period of two years prior to the pandemic (Mar 18 to Feb 20). The subsequent time during the pandemic (Mar 20 to Jun 22) was split into seven distinct time periods of four months each. This resulted in eight distinct samples from eight time spans for analysis (see Table 2). In the second analysis, we added two SES indicators as further predictors: highest educational level (primary education / secondary education / post-secondary or tertiary education) and monthly net household income (< 1300 EUR / 1300 – 2200 EUR / > 2200 EUR). Income levels were chosen based on poverty thresholds in Austria (Statista, 2022). Missing data in the HADS and PTSS-10 were imputed if a maximum of two items were missing. This was the case for 12 patients in the PTSS-10 and 19 patients in the HADS. Patients with more missing data were excluded from analysis.

**Table 2:** Mean values and standard deviation of the dependent variables upon all time periods.

	Anxiety		Depression		PTSS		Fatigue	
	*M*	*SD*	*M*	*SD*	*M*	*SD*	*M*	*SD*
Before the pandemic (Mar 18 - Feb 20)	·79	·01	·73	·02	·96	·01	·51	·01
Mar 20 - Jun 20	·80	·04	·80	·10	·98	·04	·59	·04
Jul 20 - Oct 20	·72	·03	·68	·03	·94	·03	·47	·03
Nov 20 - Feb 21	·69	·03	·60	·03	·86	·03	·47	·03
Mar 21 - Jun 21	·77	·03	·69	·03	·93	·03	·51	·03
Jul 21 - Oct 21	·78	·04	·70	·05	·95	·04	·56	·04
Nov 21 - Feb 22	·74	·04	·66	·04	·99	·03	·49	·04
Mar 22 - Jun 22	·75	·03	·65	·03	·96	·03	·53	·03

*Note.* Raw scores were log(*x* + 1)-transformed.

All four dependent variables were log(*x* + 1)-transformed due to high skewness. Transformation was shown to be a robust procedure for right-skewed data in simulation studies (Hammouri et al., 2020). Separate ANOVAs and Bonferroni pairwise comparisons were applied as post-hoc tests. Tests were two-sided and Alpha level was set to *p* < .05. No adjustments for multiple testing were made due to an individual interest in each dependent variable and therefore an individual testing approach (Rubin, 2021). For ease of graphical interpretation, plotted data in the Figures was z-transformed. This transformation standardizes each scale to a mean of zero and a standard deviation of one, ensuring direct comparability between scales. Analyses were performed using SPSS 28. Due to the nature of this study, randomization or blinding was not applicable. A power analysis was not feasible because a natural sample within the COVID-19 pandemic was analysed, without the possibility of pre-determining the sample size. The data underlying this article are available in the Open Science Framework (OSF), at https://doi.org/10.17605/OSF.IO/7THFY (Zeilinger & Gaiger, 2022).

## Results

**Fig. 1:**
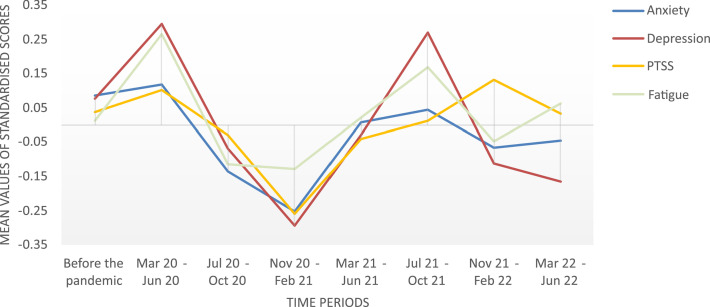
Anxiety, depression, PTSS, and fatigue before and during the pandemic. The Figure shows the psychological symptom burden before the pandemic, as well as across several time periods within the pandemic. Scores have been z-transformed to aid visual interpretation.

Scores of anxiety, depression, PTSS, and fatigue across all time periods are shown in Table 2 and Fig. 1. The first MANOVA examined the sole impact of the COVID-19 pandemic on psychological symptom scores. Therefore, timespan was included as only predictor. The MANOVA showed a statistically significant difference between the respective time periods on the combined dependent variables (F(28, 4753.54) = 1.94, P = .002, partial η² = .01, Wilk's Λ = .96). Post-hoc tests showed a statistically significant difference for anxiety (*p* = .018) and depression (*p* < .001), but not for PTSS (*p* = .106) and fatigue (*p* = .197). For anxiety, Bonferroni pairwise comparisons revealed a significant difference only between the reference period before the pandemic, and Nov 20 – Feb 21 (p = .016), with less anxiety being reported in Nov 20 – Feb 21. For depression, three significant differences were found; all related to the time span of Nov 20 – Feb 21. In this time span, depressive symptoms were significantly less reported than before the pandemic (*p* = .004), and than in the two time periods Mar 20 – Jun 20 (*p* = .008) and Jul 21 – Oct 21 (*p* = .032). All other pairwise comparisons did not show significant results.

**Fig. 2:**
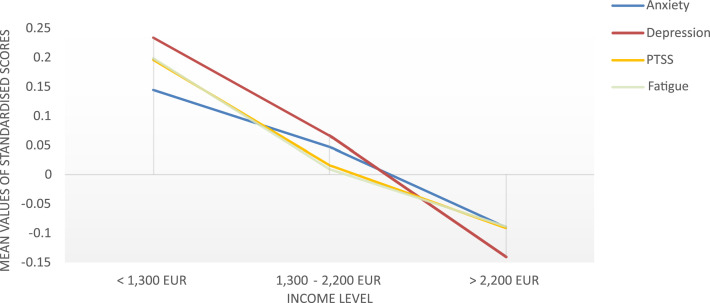
Symptoms of anxiety, depression, PTSS, and fatigue across all income levels. The Figure shows the psychological symptom burden across income levels. Scores have been z-transformed to aid visual interpretation. This transformation standardizes each scale to a mean of zero and a standard deviation of one, ensuring direct comparability between scales.

In the second MANOVA we included two SES indicators (income and education level) together with timespan as predictors in the analysis. Statistically significant differences were only found between income levels on the combined dependent variables (F(8, 2572) = 0.7, P = .023, partial η² = .01, Wilk's Λ = .99). Timespan was not a significant predictor. Post-hoc ANOVAs showed a statistically significant difference for all four dependent variables related to income. Patients with the lowest income level showed significantly higher symptom burden than patients with the highest income level in all dependent variables (*p_s_* ranging from < 0.001 to 0.004; see Table 3). The highest mean difference was found for depressive symptoms (*MD* = 0.13, *CI* = [0.07; 0.19], *p* < .001). All pairwise comparisons are depicted in Table 3. Depressive symptoms were the only dependent variable that showed a significant difference also between the group with middle income and lowest income. In Fig. 2, mean symptom burden is plotted across income levels and illustrates the high symptom burden in people with low income.

**Table 3:** Pairwise comparisons of income levels on psychological symptom burden.

		1300 EUR – 2200 EUR				> 2200 EUR		
		Mean difference	95% CI	*P*		Mean difference	95% CI	*P*
Anxiety	< 1300 EUR	0.03	[−0.03; 0.09]	0.634		**0.07**	**[0.02; 0.13]**	**0.004**
	1300 EUR – 2200 EUR	–	–	–		0.43	[−0.00; 0.08]	0.087
								
Depression	< 1300 EUR	0.06	[−0.01; 0.12]	0.09		**0.13**	**[0.07; 0.19]**	**< 0.001**
	1300 EUR – 2200 EUR	–	–	–		**0.07**	**[0.02; 0;12]**	**0.003**
								
PTSS	< 1300 EUR	0.06	[−0.00; 0.12]	0.063		**0.09**	**[0.04; 0.15]**	**< 0.001**
	1300 EUR – 2200 EUR	–	–	–		0.03	[−0.01; 0.08]	0.275
								
Fatigue	< 1300 EUR	**0.06**	**[0.01; 0.12]**	**0.045**		**0.09**	**[0.04; 0.12]**	**< 0.001**
	1300 EUR – 2200 EUR	–	–	–		0.03	[−0.02; 0.08]	0.357

*Note.* For each of the four dependent variables, all three income levels are compared with each other. Significant results are marked in bold. Positive mean differences indicate higher symptom burden in patients with lower income.

### Ad-hoc analysis on income levels

To examine a potential explanation of the findings of this study, we applied a X^2^ test to examine distribution of income levels before and during the pandemic. Results show that our data included significantly less patients with lower income during the pandemic (X^2^(2) = 37.69; *p* < .001).

## Discussion

The present study investigated the impact of the pandemic and SES indicators on psychological burden of cancer outpatients. We found that the pandemic had no impact once SES indicators were included in the analysis. Lower income was the most significant predictor of psychological distress and this effect was independent of the COVID-19 pandemic. Examining only the influence of time, we could have concluded that the pandemic had a direct influence on psychological outcomes, with patients being less distressed in the period Nov 20 – Feb 21, when a hard lockdown took place.. However, when income and education level were included as predictors in addition to timespan, the influence of the pandemic was negligible, whereas lower income was a significant predictor of higher psychological symptom burden across all dependent variables, i.e. anxiety, depression, PTSS, and fatigue. One explanation for this seemingly discrepancy in analyses is that patients with low SES, who are generally more burdened, were less likely to seek cancer care during the pandemic (Zeilinger, Lubowitzki et al., 2022). In an ad hoc analysis, we tested our data against this theory and found that during the pandemic, people with lower incomes were indeed underrepresented in our data, which may have caused the average decrease in psychological symptom burden. When we controlled for this factor in our second analysis by including SES indicators as predictors, the negligible effect of the pandemic became apparent.

The pandemic had a significant impact on mental health in different populations, especially in young adults and children, mental health problems related to the pandemic could be observed in various research efforts (Bai et al., 2022). In cancer patients, the pandemic negatively impacted health care and cancer care, especially in already undeserved groups including those with low SES (Amram et al., 2021; Zeilinger, Lubowitzki et al., 2022). However, our data indicate, that the pandemic does not have a direct impact on psychological distress in cancer patients. It may well be that the generally heavy psychological symptom burden in cancer patients cannot be further elevated by the pandemic, or that the cancer diagnosis as such is the more significant stressor. Among cancer patients, however, there are and always have been patients who are particularly vulnerable, including people of low SES. We show that poverty is a major cause of higher psychological symptom burden, also during the pandemic. A study conducted in eight European countries also indicates that in the general adult population income difficulties were an important factor contributing to anxiety and depression during the pandemic (Hajek et al., 2022), highlighting low SES as a major mental health stressor in a mixed-population group.

Sociodemographic characteristics were also found to have a significant impact on emotional response among health care workers, with those with children showing a higher increase of emotional exhaustion over time (Maunder et al., 2021). Therefore, the emotional and psychological response to the COVID-19 pandemic of different groups of people may generally be mediated by a person's individual characteristics. This could also contribute to inconsistent results found in existing research if such individual characteristics are not taken into account or are controlled for in the analysis.

It should be noted that our data was collected at the Vienna General Hospital, the largest public oncology care center in Austria. The health care system in Austria provides health insurance to every person, regardless of employment status. Therefore, anyone can access oncology care at our center and our sample is not biased by insurance status.

Given the strong impact of psychiatric comorbidities on overall survival in cancer patients (Gaiger et al., 2022), there is a need to provide low-threshold, affordable psychosocial care for patients with low income. We also point out, that research efforts examining the impact of the pandemic would be erroneous if other potential influencing factors were not considered. An exclusive focus on the pandemic could be one aspect contributing to the diverging results of studies on this topic.

### Strengths and limitations

One strength of this research lies in the standardized routine assessment of psychological distress before and during the COVID-19 pandemic, which provides the opportunity to reliably compare psychological distress across these time periods. However, participation in this study was voluntary. Therefore, we have no influence on sample characteristics, which can bias our results. This also includes the impact of cancer type and severity of the illness. Certain cancer types are associated with low SES and poor health behaviours, such as aggressive ENT tumours, which are associated with the risk factors of alcoholism and smoking. We did not control for cancer type and therefore cannot make assumptions about the impact of the pandemic on patients with specific cancers or illness severity. However, our sample is an unselected mixed cancer sample found at the largest public outpatient clinic in Austria and therefore has high external validity. Another limitation of this study is the use of screening instruments. Although we cannot make statements on psychiatric diagnoses by means of these instruments, they are well-suited to assess psychological symptom burden.

We are aware, that there are multiple factors influencing psychological well-being, and that we only included some of the most prominent aspects, SES indicators, in the analysis. Further aspects, including physical symptom burden, cancer stage, or treatment phase were not included. Yet, considering sample size and statistical power, it would not have been advisable to include more predictors in the present analysis. As a single-center study, the present work is exploratory in nature and needs to be confirmed in other settings or centres.

### Conclusion

Since the pandemic has taken hold of the world, it is omnipresent in every part of life, including health care and research. In many respects this is justified, as this novel situation poses innumerable challenges and obstacles that need to be addressed. However, the pandemic is not the only threat that impacts health, health care, psychological wellbeing, and survival. While we are trying to find ways to support patients during the pandemic, which is undoubtedly an essential task, it may also be worthwhile to focus on the individual needs of patients who require psychosocial support because of their cancer diagnosis and relevant risk factors such as poverty, rather than because of the pandemic.

## Financial support

This research received no specific grant from any funding agency, commercial or not-for-profit sectors.

## Preprint information

A preprint of this manuscript is available in MedRxiv at https://doi.org/10.1101/2022.11.21.22282580

## Declaration of Competing Interest

The authors declare the following financial interests/personal relationships which may be considered as potential competing interests:

**Rupert Bartsch:** Consulting fees: Astra-Zeneca, Daiichi, Eisai, Eli-Lilly, Gilead, Gruenenthal, MSD, Novartis, Pfizer, Pierre-Fabre, Puma, Roche, Seagen. Lecture Honoraria: Astra-Zeneca, Daichi, Eisai, Eli-Lilly, Gilead, Gruenenthal, MSD, Novartis, Pfizer, Pierre-Fabre, Roche, Seagen. Support for attending meetings and/or travel: AstraZeneca, Daiichi, MSD, Roche.

**Peter Valent**: Consulting fees: Novartis, Blueprint, BMS/Celgene, Pfizer, Cogent, and AOP Orphan.

**All other authors** have nothing to disclose.

## Data Availability

The data underlying this article are available in the Open Science Framework (OSF), at https://doi.org/10.17605/OSF.IO/7THFY
